# Epidemiology of hip fractures in Costa Rica and development of country- specific thresholds to estimate fracture risk

**DOI:** 10.1007/s11657-026-01659-z

**Published:** 2026-02-03

**Authors:** S. Cerdas Perez, Griselda-Adriana Cruz-Priego, Patricia Clark, Adolfo Ortiz-Barboza, H. Johansson, J. A. Kanis

**Affiliations:** 1 Department of Endocrinology, Hospital Cima San José, Universidad de Costa Rica, San José, Costa Rica; 2 Clinical Epidemiology Research Unit, Hospital Infantil de Mexico “Federico Gomez”, Mexico City, Mexico; 3 Faculty of Medicine, National Autonomous University of Mexico (Universidad Nacional Autónoma de México), Mexico City, Mexico; 4 University of Costa Rica, San José, Costa Rica; 5 Centre for Metabolic Bone Diseases, University of Sheffield Medical School, Beech Hill Road, S10 2RX, Sheffield, UK; 6 Mary McKillop Health Institute, Australian Catholic University, Melbourne, Australia

**Keywords:** FRAX®, Hip fracture, Osteoporosis, Fragility fracture, Fracture risk, Costa Rica

## Abstract

***Summary*:**

A country-specific FRAX® model for Costa Rica was developed using national hip fracture data from 2015–2019. Hip fracture incidence increased with age, was consistently higher in women, and exceeded rates reported in neighboring Latin American countries. Costa Rica also demonstrated higher 10-year major osteoporotic fracture probabilities at older ages compared with regional FRAX® models. An estimated 3,176 hip fractures occurred in 2020, with projections indicating an increase to 9,946 cases by 2050. These findings underscore the growing burden of hip fractures in Costa Rica and the need for targeted prevention strategies.

**Objective:**

To describe the epidemiology of hip fractures in Costa Rica and to develop a country-specific FRAX® model calibrated with national fracture and mortality data.

**Methods:**

All hip fractures (ICD-10 S72.0, S72.1, S72.2) recorded in the Caja Costarricense de Seguro Social registry from 2015–2019 were included. Age- and sex-specific incidence rates were combined with United Nations mortality data to construct a Costa Rica–specific FRAX® model. Ten-year fracture probabilities were estimated and compared with those from other Latin American countries with calibrated FRAX® models. National projections for hip fracture burden in 2020 and 2050 were derived using United Nations population forecasts. Age-specific FRAX® assessment and intervention thresholds were developed following established international methodology.

**Results:**

Hip fracture incidence increased with age and was consistently higher in women than in men. Compared with other Latin American countries, Costa Rica demonstrated higher age-specific hip fracture rates and higher 10-year major osteoporotic fracture probabilities at older ages. An estimated 3,176 hip fractures occurred in adults aged ≥ 50 years in 2020, with a projected increase to 9,946 by 2050. Age-specific FRAX® threshold curves showed widening separation with advancing age, reflecting increasing fracture probability in older age groups.

**Conclusion:**

Costa Rica exhibits a comparatively high burden of hip fractures and fracture risk, which is expected to rise substantially with population aging. The newly developed Costa Rica–specific FRAX® model provides an essential tool for improving fracture risk assessment and guiding evidence-based osteoporosis management in clinical practice.

## Introduction

Osteoporosis is a progressive skeletal disorder characterized by reduced bone strength and an increased risk of fracture [1]. Fragility fractures represent a major global health burden, leading to substantial disability, reduced quality of life, and elevated mortality [2]. Although dual-energy X-ray absorptiometry (DXA) remains the reference method for diagnosing osteoporosis, access to DXA is limited in many regions, particularly in low- and middle-income countries. In this context, validated fracture risk prediction tools that can be applied with or without bone mineral density (BMD) have become essential for clinical decision-making [3].

FRAX® is a computer-based algorithm that estimates the 10-year probability of major osteoporotic and hip fractures using clinical risk factors, with optional inclusion of BMD [4]. Since its launch in 2008, FRAX® has become an international standard for fracture risk assessment. However, fracture probability varies widely across populations due to differences in epidemiology and mortality; thus, each FRAX® model must be calibrated with country-specific hip fracture incidence and mortality data to ensure accuracy and applicability [5].

Within Latin America, fully calibrated FRAX® models are available for only seven countries—Argentina, Brazil, Chile, Colombia, Ecuador, Mexico, and Venezuela—and a surrogate model exists for Peru, based on epidemiologic similarities with calibrated countries [6]. The absence of a Costa Rica–specific model limits the ability of clinicians to estimate fracture risk using local epidemiological patterns.

The aim of this study was to describe the epidemiology of hip fractures in Costa Rica and to develop a country-specific FRAX® model calibrated with national fracture incidence and mortality data.

## Methods

Hip fracture data were obtained from the hospital discharge database of the Caja Costarricense de Seguro Social (CCSS), the principal public health care provider in Costa Rica. The CCSS covers approximately 91% of the national population and is therefore considered representative at the country level. All cases recorded between 2015 and 2019 with a primary diagnosis coded as ICD-10 S72.0 (femoral neck fracture), S72.1 (pertrochanteric fracture), or S72.2 (subtrochanteric fracture) were included. These codes correspond to the internationally accepted definitions of hip fractures used in the development and calibration of FRAX® models.

Age- and sex-specific incidence rates for hip fracture were calculated using population estimates from the National Institute of Statistics and Census (INEC). To construct the Costa Rica–specific FRAX® model, these incidence rates were combined with national mortality data obtained from the United Nations (UN) population and mortality datasets for 2015–2019 [11]. Mortality inputs were incorporated following standard FRAX® computational procedures to generate 10-year probabilities of hip fracture and major osteoporotic fracture.

Ten-year fracture probabilities presented in Table 2 were generated using the FRAX® computational engine populated with Costa Rican epidemiological inputs. Probabilities were estimated for both sexes by age and body mass index (BMI), assuming the absence of clinical risk factors. Major osteoporotic fracture probabilities were derived from established FRAX® algorithms that relate hip fracture incidence to the incidence of other major skeletal sites (spine, forearm, and humerus).

To estimate the national number of hip fractures in 2020 and 2050, the mean incidence rates from 2015–2019 were applied to UN population projections for those years. The year 2020 was selected as the initial projection point because it represents the earliest year with complete population estimates following the incidence period, thus enabling standardized comparisons with projection methodologies used in other calibrated FRAX® models.

Assessment and intervention thresholds were constructed following the case-finding methodology proposed by Kanis et al. Using the Costa Rica–specific FRAX® model without BMD, age-specific 10-year probabilities of major osteoporotic fracture were calculated for a woman with a BMI of 25 kg/m^2^ under three clinical profiles: a woman without clinical risk factors, a woman with a prior fragility fracture, and the latter value multiplied by 1.2 to define a very-high-risk threshold. The use of 1.2 times the intervention threshold aligns with the recommendation by Kanis et al. [7], who propose setting the upper assessment threshold at 20% above the intervention threshold to identify individuals whose fracture risk warrants immediate treatment without further evaluation [8]. These values were plotted across ages to generate continuous curves representing the lower assessment threshold, the intervention threshold, and the upper assessment threshold. When displayed graphically, these curves allow stratification of individuals into low, intermediate, high, and very high fracture risk categories, providing an age-specific FRAX®-based framework for evaluation and clinical decision-making in the Costa Rican population.

Finally, hip fracture incidence in Costa Rica was compared with that of other Latin American countries with calibrated FRAX® models, including Mexico, Venezuela, Colombia, and Ecuador. Comparisons included both age-specific incidence curves and corresponding 10-year FRAX® fracture probability estimates.

## Results

Age- and sex-specific incidence rates of hip fractures in adults aged ≥ 50 years from 2015 to 2019 are shown in Table 1. Incidence increased progressively with age in both sexes, with higher rates observed in women across nearly all age groups. In men, the most marked increases occurred in the 65–69 and 75–79 age groups, each showing increases of just over 20%. In women, rises were more moderate but consistent, particularly between ages 65–69 (14%) and 75–79 (11%).

**Table 1:** Age-specific incidence of hip fractures per 100,000 population in Costa Rica, by sex

	Year	Year period							
		50–54	55–59	60–64	65–69	70–74	75–79	80–84	> 85
Men	2015	45.7	56.1	63.7	106.0	161.3	290.4	610.4	1505.5
	2016	37.3	58.2	64.5	106.1	196.0	313.1	642.9	1405.8
	2017	46.1	60.1	83.9	95.4	168.7	318.3	797.1	1660.5
	2018	52.8	50.3	76.5	99.6	163.8	271.5	595.9	1499.7
	2019	52.3	53.0	72.9	131.2	156.8	351.3	680.6	1443.5
Women	2015	33.7	53.1	111.6	184.2	359.1	596.7	1125.4	2139.2
	2016	29.0	56.1	118.2	169.2	350.5	597.2	1124.7	2369.1
	2017	35.4	60.3	125.6	202.1	313.6	658.9	1222.2	2263.4
	2018	29.9	50.4	118.8	175.2	281.2	640.5	1137.5	2175.9
	2019	34.5	55.3	107.9	210.9	335.7	663.3	1097.7	2092.7

**Fig. 1:**
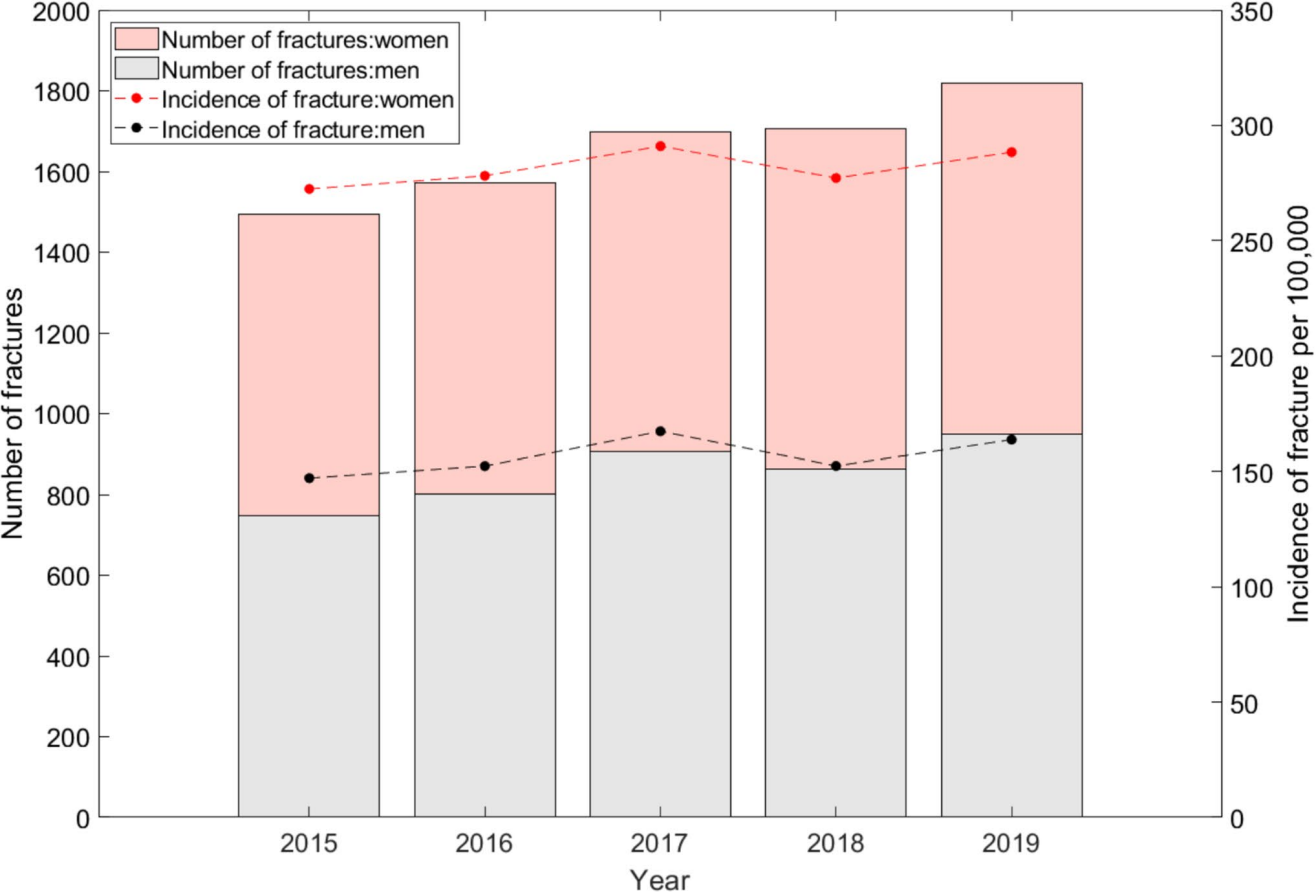
Combined graph of incidence and number of hip fractures in Costa Rica (2015–2019)

Figure 1 displays annual hip fracture incidence and the total number of fractures by sex from 2015 to 2019. Throughout the observation period, women experienced nearly twice as many fractures as men, and the number of events increased progressively in both sexes. Using the mean incidence rates from 2015–2019 and applying them to United Nations population estimates, we estimated that 3,176 hip fractures occurred in Costa Rica in 2020 among adults aged ≥ 50 years. Based on projected demographic changes, this number is expected to increase approximately 3.1-fold to 9,946 hip fractures by 2050.

Table 2 presents 10-year major osteoporotic fracture probabilities estimated using the Costa Rica–specific FRAX® model. As expected, fracture probability increased steadily with age and was consistently higher in women than in men. Across all ages, higher BMI values were associated with lower fracture probabilities.

**Table 2:** Ten-year probability of osteoporotic fracture by age and body mass index (BMI) without other clinical risk factors

10−year probability of osteoporotic fracture without clinical risk factors							
Age (years)	Body mass index						
	15	20	25	30	35	40	45
Men							
50	2.5	2.3	2.3	2.0	1.7	1.5	1.3
55	2.9	2.7	2.6	2.2	1.9	1.7	1.5
60	3.6	3.2	3.0	2.6	2.2	1.9	1.7
65	4.4	3.8	3.4	2.9	2.5	2.1	1.8
70	5.7	4.8	4.2	3.6	3.0	2.6	2.2
75	7.7	6.5	5.6	4.7	3.9	3.3	2.8
80	9.4	8.1	7.0	5.8	4.8	3.9	3.2
85	11	9.5	8.3	6.7	5.4	4.4	3.6
90	12	10	9.3	7.4	5.8	4.6	3.7
Women							
50	3.4	3.0	2.9	2.5	2.2	1.9	1.7
55	4.4	3.9	3.6	3.1	2.7	2.4	2.1
60	6.0	5.1	4.5	3.9	3.4	3.0	2.6
65	7.9	6.4	5.6	4.8	4.1	3.6	3.1
70	11	8.8	7.5	6.4	5.5	4.7	4.0
75	16	13	11	9.1	7.7	6.6	5.6
80	21	17	14	12	10	8.6	7.2
85	24	21	18	15	12	10	8.5
90	25	23	20	16	13	11	8.8

**Fig. 2:**
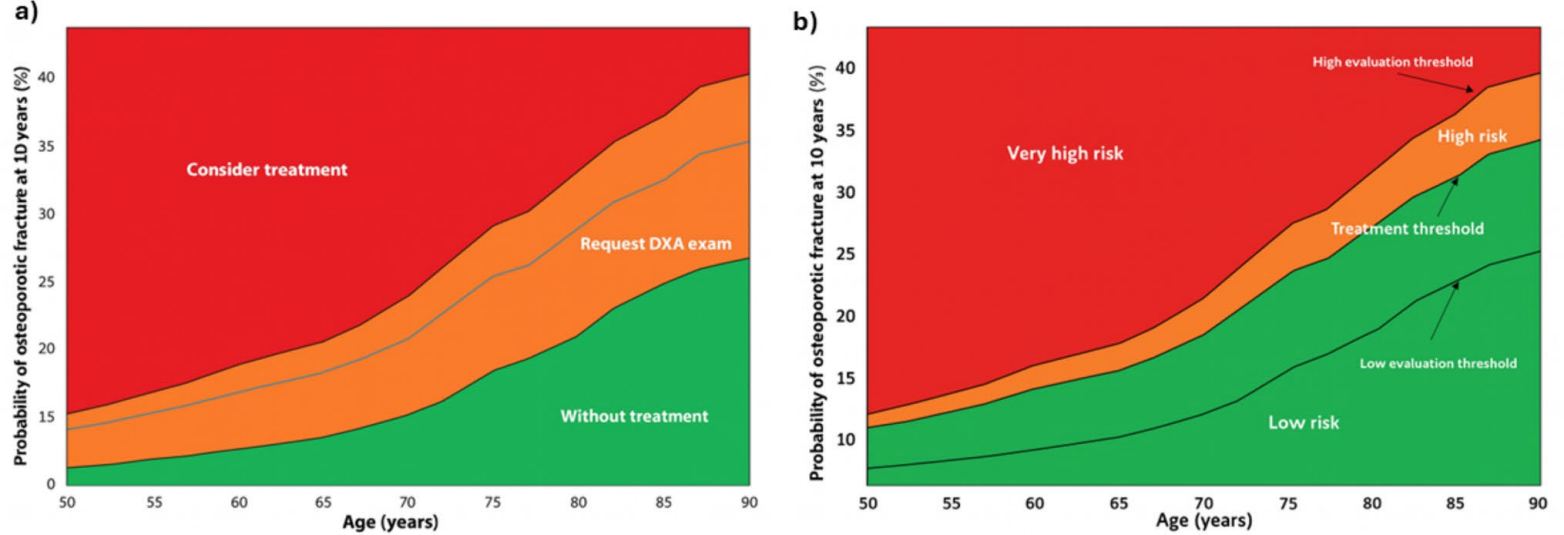
**a**) FRAX® assessment thresholds are used in primary care to identify patients at risk of fracture. In the Costa Rican population. **b**) FRAX® intervention thresholds are used to guide treatment decisions for patients

Assessment and intervention thresholds derived from the Costa Rica FRAX® model are shown in Fig. 2. The graphical representation demonstrates that fracture probability rises markedly with age, with widening separation between the lower assessment threshold, intervention threshold, and upper assessment threshold in older age groups. These age-specific thresholds allow categorization of individuals into low, high, and very high risk strata.

**Fig. 3:**
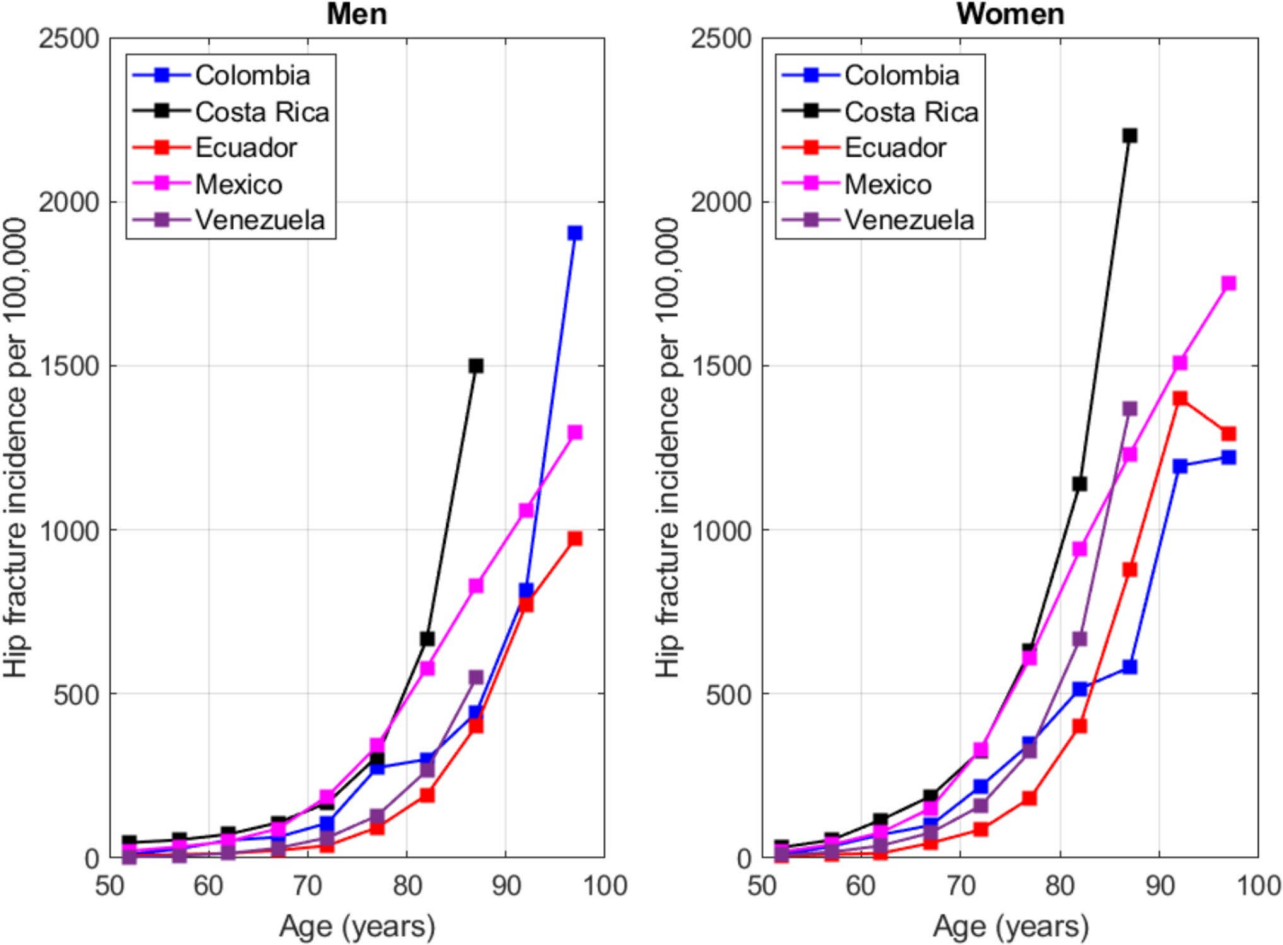
Incidence of hip fracture in Costa Rica by age and comparison with other Latin American countries (Mexico, Venezuela and Colombia)

Figure 3 compares age-specific hip fracture incidence in Costa Rica with that of Mexico, Venezuela, and Colombia. Across most age groups, Costa Rica exhibited higher hip fracture incidence in both sexes, suggesting a relatively greater burden of hip fractures in the country compared with neighboring Latin American nations.

**Fig. 4:**
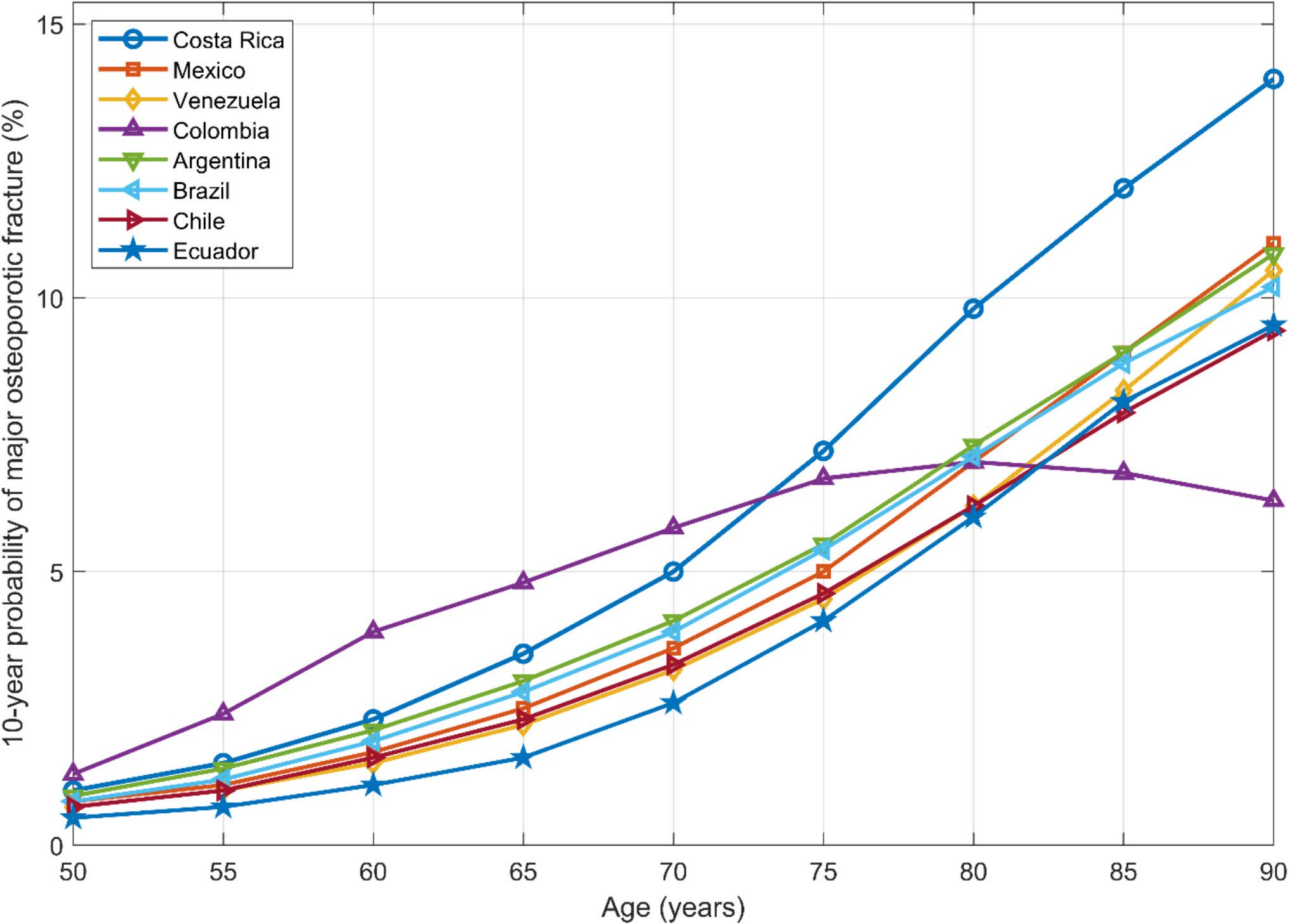
Ten-year probability of a major osteoporotic fracture (MOF) by age in women (BMI 25 kg/m^2^, no clinical risk factors), comparing Costa Rica with other Latin American countries with calibrated FRAX® models

Finally, Fig. 4 illustrates the 10-year probability of major osteoporotic fracture across age for all available Latin American countries with calibrated FRAX® models, including Costa Rica. The Costa Rica curve showed higher probabilities than most countries at older ages, reinforcing the comparatively high fracture risk in the Costa Rican population and supporting the need for targeted prevention strategies.

## Discussion

The present study provides the first nationally calibrated FRAX® model for Costa Rica and describes population-based hip fracture incidence in adults aged ≥ 50 years. As reported globally, hip fracture incidence was substantially higher in women than in men [8, 9], consistent with the well-established sex differences in osteoporosis prevalence and fracture risk. The progressive increase in incidence observed between 2015 and 2019 aligns with international data showing a continuously rising burden of fragility fractures in aging populations [6].

Our projections indicate that the number of hip fractures in Costa Rica will increase more than threefold by 2050. This trend is driven largely by demographic aging, a pattern seen across Latin America, where approximately 23% of the population is now ≥ 50 years old [10]. Similar increases have been documented in other countries undergoing rapid demographic transition, including Brazil [12].

When Costa Rica was compared with other Latin American countries using available calibrated FRAX® models (Fig. 4), Costa Rican women demonstrated higher 10-year major osteoporotic fracture probabilities at older ages. This pattern parallels the higher age-specific hip fracture incidence observed relative to Mexico, Venezuela, and Colombia, suggesting that Costa Rica carries a comparatively greater burden of fragility fracture risk within the region. These findings underscore the importance of developing prevention strategies tailored to the country’s demographic and epidemiological profile.

Ethnic and genetic heterogeneity may also influence fracture risk in Costa Rica. Several studies have demonstrated significant differences in bone mineral density, bone geometry, and fracture patterns among ethnic groups [13–15]. Costa Rica’s admixed population—comprising Indigenous, European, and African ancestries—may therefore exhibit fracture risk patterns distinct from other Latin American countries. Lifestyle and environmental factors such as diet, physical activity, vitamin D status, and healthcare accessibility likely contribute further to these differences.

The availability of a Costa Rica–specific FRAX® model represents a major advance for national clinical practice. FRAX® is the only fracture prediction tool calibrated to country-specific fracture and mortality rates, and its implementation has been associated with improved identification of high-risk individuals and reductions in hip fracture incidence when used in screening strategies [16]. Given the limited accessibility to DXA scanning in some regions of Costa Rica, FRAX® offers a practical and evidence-based method for risk assessment and treatment decision-making. The use of age-specific assessment and intervention thresholds, consistent with current international guidelines [17], allows clinicians to classify individuals into low, high, or very high risk categories and to target treatment to those who may benefit most.

Nevertheless, FRAX® has limitations. Several risk factors are not included in the algorithm, and some are incorporated in qualitative rather than quantitative form [18]. Newer tools such as FRAXplus® aim to address these issues by integrating additional predictors and improving accuracy [18]. Further research in Costa Rica will be essential to refine fracture risk assessment and deepen understanding of local epidemiological patterns.

Several limitations of this analysis should be considered when interpreting these findings. As with all FRAX® calibrations, absolute fracture risk estimates depend on the quality and completeness of national fracture incidence and mortality data. First, fracture incidence estimates were derived from administrative hospital discharge records, which do not include detailed clinical information at the individual level. Second, national projections for 2020 and 2050 assume that age- and sex-specific hip fracture incidence rates observed during 2015–2019 remain stable over time; therefore, future changes in osteoporosis management, fracture prevention strategies, or healthcare access could modify these estimates. Finally, regional comparisons were limited to Latin American countries with calibrated FRAX® models and publicly available data, which may not fully capture the heterogeneity of fracture risk across the region.

Overall, our findings underscore the growing burden of fragility fractures in Costa Rica and highlight the need for targeted strategies, including improved access to osteoporosis evaluation, expansion of preventive services, and implementation of coordinated post-fracture care programs such as Fracture Liaison Services.

## Conclusion

Costa Rica exhibits a progressively increasing incidence of hip fractures, particularly after age 70, with consistently higher rates in women compared with men. Based on demographic projections, the number of hip fractures among adults aged ≥ 50 years is expected to rise more than threefold by 2050, representing a substantial and growing burden for the national healthcare system. The higher fracture risk observed in Costa Rican women at older ages compared with other Latin American countries further underscores the need for coordinated prevention strategies. The development of a Costa Rica–specific FRAX® model provides an essential tool for improving fracture risk assessment, particularly in settings where access to densitometry is limited. Incorporating this calibrated model into routine clinical practice may enhance early identification of individuals at high or very high risk, support more efficient use of DXA resources, and guide evidence-based treatment decisions aimed at reducing the future impact of osteoporotic fractures in the country.

### Future directions

Several actions could strengthen the clinical impact of the Costa Rica FRAX® model. First, dissemination and implementation strategies are needed to promote its routine use in primary and specialized care, with or without bone mineral density testing. Second, locally validated age-specific intervention and assessment thresholds should be further refined to support optimized clinical decision-making. Third, expanding post-fracture care programs, including the development of Fracture Liaison Services (FLS), could substantially improve secondary fracture prevention nationwide. Finally, greater emphasis on osteoporosis as a public health priority—through education, training, and research—will be essential to understanding evolving fracture trends and improving outcomes for the Costa Rican population.

## Data Availability

All data generated or analyzed during this study are fully reported within the manuscript.
